# Evaluation of Antioxidant Capacity of* Solanum sessiliflorum* (Cubiu) Extract: An* In Vitro* Assay

**DOI:** 10.1155/2015/364185

**Published:** 2015-12-15

**Authors:** Diego Rocha de Lucena Herrera Mascato, Janice B. Monteiro, Michele M. Passarinho, Denise Morais Lopes Galeno, Rubén J. Cruz, Carmen Ortiz, Luisa Morales, Emerson Silva Lima, Rosany Piccolotto Carvalho

**Affiliations:** ^1^Programa Multi-Institucional de Pós-Graduação em Biotecnologia, Universidade Federal do Amazonas, 69077-000 Manaus, AM, Brazil; ^2^Division of Biochemistry, Ponce Health Sciences University, School of Medicine, Ponce Research Institute, Ponce, PR 00732-7004, USA; ^3^Biology Department, University of Puerto Rico at Ponce, Ponce, PR 00734, USA; ^4^Divisions of Pharmacology & Toxicology & Cancer Biology, Ponce Health Sciences University, School of Medicine, Ponce Research Institute, Ponce, PR 00732-7004, USA; ^5^Public Health Program, Ponce Health Sciences University, Ponce, PR 00732-7004, USA; ^6^Faculdade de Ciências Farmacêuticas, Universidade Federal do Amazonas, 69010-300 Manaus, AM, Brazil

## Abstract

Cubiu is a vegetable of Solanaceae family, native to the Amazon, which is widely distributed through Brazil, Peru, and Colombia. It is used in food, medicine, and cosmetics by native populations. Research has shown that cubiu extracts have antioxidant activities with great biological relevance. We performed a phytochemical screening to identify the main chemical groups that could confer antioxidant activity to this extract. Several tests and qualitative precipitation specific staining for major classes of secondary metabolites were used. Antioxidant capacity* in vitro* tests (DPPH and ABTS) were also used to assess the extract's ability to sequester free radicals of 70% hydroethanolic and aqueous extracts of cubiu flour. Alkaloids, organic acids, phenols, flavonoid glycosides, and coumarins were found in the hydroethanolic extract while the aqueous extract presented anthocyanins, gums, tannins and mucilage, amino groups, and volatile and fixed acids. For* in vitro* tests, the IC_50_ value obtained in the DPPH assay was 606.3 ± 3.5 *μ*g/mL while that for the ABTS assay was 290.3 ± 10.7 *µ*g/mL. Although cubiu extracts present chemical compounds directly related to antioxidant activity, our results show that it has a low antioxidant activity. Additional studies will be needed to isolate and characterize specific compounds to further assess antioxidant activity.

## 1. Introduction

Cubiu (*Solanum sessiliflorum* Dunal), also known in Spanish-speaking countries as “tupiro,” “topiro,” and “cocona” and known as “Orinoco apple” and “peach tomato” in English-speaking countries, is a vegetable of the family Solanaceae which is native to the Amazon [6] and widely distributed through the humid equatorial regions of Brazil, Peru, and Colombia. Its domestication was made by the Indians of Western Amazonia [7]. The fruit can weigh between 20 and 450 grams and contains between 200 and 500 oval flat seeds. The fruits have the most diverse forms. Those cylindrical in shape have generally 4 loci, while the heart-shaped, round, and flattened ones have between 6 and 8 loci. The fruit color changes as it matures, ranging from green when it is not ripe to yellow-orange when it is mature and finally to red when coffee is ready to be consumed. The fruit has a pulp color ranging from light yellow to yellowish cream, measuring between 0.2 and 2.5 cm thick [6].

The pulp is the edible part of the fruit. As food, it is consumed “naturally” as strip-like alcoholic beverages, jellies, jams, juices, and sauces [1, 8]. The macerated leaves of the plant are used by Peru-Brazil indigenous people to avoid the formation of blisters in the event of skin burns, while the juice from the fruit's locular cavity is used to alleviate the itching of spider bites [9]. The pure juice is used for the control of cholesterol, diabetes, excess uric acid, and other affections caused by kidney and liver failure, besides being used to eliminate lice [9]. Previous studies have shown the composition of cubiu as presented in Table 1.

Cubiu is a juicy fruit with humidity between 88 and 93%. It has a variable soluble solids content (°Brix) between 5 and 8, of which the majority are reducing sugars [1]. Since the ratio of °Brix and acidity of the fruit is low, it is considered acidic. This ensures a high dilution factor (great for juice production) and little preference for fresh consumption (due to sourness of the fruit). The composition of phenolic compounds is also low [1]. Taking into consideration the nutritional value of cubiu (Table 2), it presents a high dietary potential due to its low caloric and high fiber content. This supports its indication in the diet of hypercholesterolemic and hyperglycemic patients [2].

In a study conducted in Norbert Wiener University in Lima (Peru), it was found that administration of cubiu extract (40 mL/day) for three days decreased the level of LDL, triglycerides, serum cholesterol, and glucose. However, it increased serum HDL levels in 100 volunteers of both sexes [10]. Furthermore, it was found that the fruit has a phenolic compound called tannin. Phenolic compounds are products of secondary plant metabolism which are essential for their growth and reproduction. Tannins are formed under conditions of stress such as infection, injury, and UV radiation [11]. These also act as natural second class antioxidants [12]. According to their mode of action, antioxidants can be classified into primary and secondary. Primary antioxidants act by donating hydrogen or electrons to free radicals, therefore, converting them into stable products and/or reacting with free radicals, forming lipid-antioxidant complexes that can react with another free radical. Secondary antioxidants act by delaying the initiation step of autooxidation by different mechanisms including metal complexation, sequestration of oxygen, decomposition of hydroperoxides to form nonradical species, absorption of UV light, or deactivation of singlet oxygen species [13].

The phenolic antioxidants interact preferentially with the peroxyl radical due to its prevalence in the autooxidation step [15]. This mechanism of action present in plant extracts plays an important role in reducing lipid oxidation in tissues, plants, and animals because it preserves food quality and reduces the risk of developing diseases such as arteriosclerosis and cancer [16, 17]. Since antioxidants are of great relevance for the field of medicine today, there is a need to investigate the antioxidant potential of cubiu and to elucidate the role of this fruit in the diet of the population.

## 2. Material and Methods

### 2.1. Feedstock

For this study, cubiu fruits used were obtained from the Horticultural Experimental Station Alejo von der Pahlen from the National Institute of Amazonian Research (NIAR) located in Manaus, AM. The fruits were transported in coolers to the Laboratory of Food and Nutrition at the NIAR where they were processed to obtain cubiu flour as described by da Silva Filho et al. (2005) [18].

### 2.2. Preparation of Cubiu Flour

**Figure 1:**
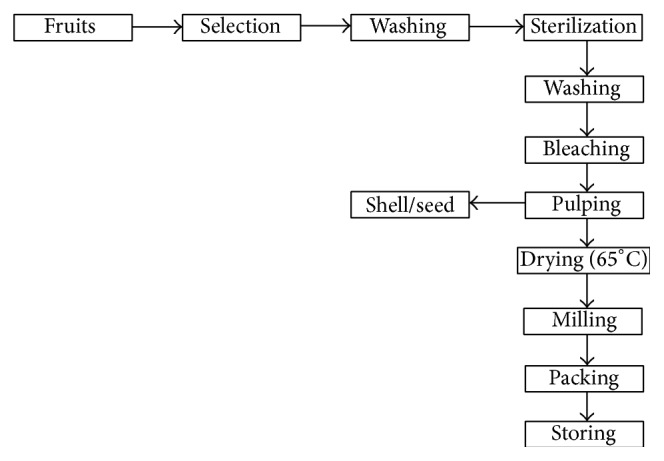
Processing of cubiu fruits to obtain the flour as described by da Silva Filho et al. [18].

After the fruits were received, the ripe intact fruits (free of cuts, holes, and other defects) were selected for processing. Fruits were washed, brushed, sanitized using a 200 ppm hypochlorite solution for 30 minutes, and rinsed with running water. After this process, the fruits were stripped of their pulp and had the placenta (inner part of the fruit that contains the seeds and the juice of the locular cavity) and the epicarp being removed with a stainless steel knife. Thermal bleaching was then performed at 90°C for 3 minutes, followed by a heat shock in an ice bath for 3 minutes. Bleached fruits were packed in 2 kg polyethylene bags and frozen at −15°C. The fruits were then dried in an oven with forced air circulation at 60°C for 48 hours. After drying, the fruits were liquified, homogenized, packed, and frozen at −15°C until they were grinded to obtain the flour. The flour was packed in plastic bags and stored in a freezer with temperature −15°C until the extract was prepared (Figure 1).

### 2.3. Preparations of Cubiu Flour Extracts

Cubiu extracts were prepared by dissolving 2 kg of cubiu flour in 2 L of ethanolic solvent. The solvent was prepared using 500 mL of distilled water and 1500 mL of ethyl alcohol (Sigma, MO). Mixtures were then placed on the Ultrasonic Cleaner (UNIQUE, Brazil) for 15 minutes to complete homogenization and filtered using filter paper and a disposable glass funnel three times. Then, the extracts were vacuum filtered using a Buchner funnel and an Erlenmeyer filtration flask. Subsequently, the extracts were placed in round bottom flasks for rotoevaporation with a 25°C water bath. The extracts were returned to their viscous form and then freeze-dried using a lyophilizer. After this, they were kept in a freezer at −11°F for later use as described by Rogério (2006) [19].

### 2.4. Assessment of Antioxidant Capacity

#### 2.4.1. DPPH Method

The DPPH assay is based on the reduction of 2,2-diphenyl-1-picrylhydrazyl radical (DPPH) through the donation of a hydrogen atom of the test compound to the radical of the molecule [20]. Measurement of DPPH radical scavenging activity was performed according to the methodology described by Molyneux (2004) [21] with some modifications. Initially, 2 mg of DPPH was dissolved in 12 mL of absolute ethanol. In a flat bottom 96-well plate, 270 *μ*L of DPPH solution was mixed with 30 *μ*L ethanol. Thereafter, the test consisted of 270 *μ*L of the solution of DPPH mixed with 30 *μ*L of 70% hydroethanolic cubiu extract. The reaction was incubated for 30 minutes at room temperature in the absence of light. After incubation, the absorbance was read at 517 nm. To express antioxidant activity IC_50_ was calculated, that is, the minimum concentration required to reduce the initial DPPH reaction by 50%. Ascorbic acid was used as standard. The antioxidant activity was calculated using the following formula:

(1) $$\begin{array}{c} \text{\%Inhibition}=100-\left(\;\frac{\text{Abs sample}}{\text{Abs control}}\;\right)\times 100. \end{array}$$

#### 2.4.2. ABTS Method

This method is based on the reduction of ABTS (2,2′azinobis-3-ethylbenzothiazoline-6-sulfonic acid) and was performed as described by Re et al. (1999) [22] with modifications. ABTS was dissolved in MiliQ water to a 7 mM concentration. ABTS^∙+^ was produced by reacting ABTS stock solution with 5 mM potassium persulfate and kept in the dark at room temperature for 12–16 h. Once ABTS^∙+^ is formed, MiliQ water was added to the solution (dilution 1 : 7). A 96-well plate flat-bottomed was added by 270 *μ*L of ABTS solution with 30 *μ*L of water. This solution was monitored by reading at 714 nm in a microplate reader (DTX 800, Beckman, CA, USA) to obtain absorbance of approximately 1.00 (control). Then, 30 *μ*L of cubiu extract from different concentrations was added to 270 *μ*L of ABTS^∙+^ and the reaction was incubated for 15 min in the dark at room temperature. After incubation, the absorbance at 714 nm was measured. Gallic acid in the same cubiu extract concentrations was measured following the same procedures described above and was used as positive controls. The antioxidant activity was calculated using the following equation:

(2) $$\begin{array}{c} \text{\%Inhibition}=100-\left(\;\frac{\text{Abs sample}}{\text{Abs control}}\;\right)\times 100. \end{array}$$

### 2.5. Systematic Analysis in Phytochemistry

This assay analyzes all the qualitative features of the principal chemical groups active in natural products, by means of staining or precipitation reactions. The systematic testing for phytochemical analysis was performed according to Moreira (1979) using maceration, aqueous extraction, and hydroethanolic extraction [23].

### 2.6. Preparation of Aqueous and Hydroethanolic Cubiu Extract

The extracts were performed by dissolving 40 g of the powder of dried cubiu fruits in 200 mL of 70% ethyl alcohol or water. The mixture was placed in an ultrasound at 50°C for 10 min. The mash was filtered through filter paper and supplemented with the same solvent volume to 200 mL. The extract was kept refrigerated until completion of the phytochemical assays.

### 2.7. Assessment for the Presence of Alkaloids, Organic Acids, and Phenols

Experiments were conducted using general alkaloid reagents (Mayer, Dragendorff, Bouchardat, and Bertrand as described in Table 4) as follows: 50 mL of the hydroethanolic extract was evaporated to dryness in a water bath at 70°C, followed by dissolution of the residue in 1 mL of ethanol and 20 mL of 1% HCl. Extracts were transferred to test tubes and the reagent was added on each one. The appearance of precipitate indicated a positive reaction. To retest, 15 mL of the aqueous extract was transferred to a separating funnel and alkalized with ammonium hydroxide (pH 10). An ether/chloroform (3 : 1) extraction was performed and the extract obtained was also tested for alkaloids. The remaining solution prepared for the study of alkaloids was dried and redissolved in 5 mL of distilled water. The pH of the solution was measured, where an acidic pH indicated the presence of organic acids. Two drops of 1% FeCl_2_ aqueous solution were added to the solution obtained in the study of organic acids, to confirm the presence of phenols.

### 2.8. Assessment for the Presence of Flavonoid Glycosides: Oxalic-Boric Complex (R. Wilson-Tauböck), Pacheco, and Shinoda Tests

10 mL of each of the extractions was dried using a water bath. Five drops of acetone and 30 mg of a mixture of boric acid and oxalic acid at a ratio of 1 : 1 were added to the dried extracts. After stirring and drying the mixture, 5 mL of ethyl ether was added and the solution was transferred to test tubes. Solutions were seen under UV light. The reaction is considered positive when the result has a greenish yellow fluorescent appearance. For the Pacheco test, a few crystals of NaOAc and 100 *μ*L of acetic anhydride were added to the dried extract. The mixture was heated in a water bath and 100 *μ*L of concentrated HCl was added. The result of the reaction is considered positive when there was purple coloring. For the Shinoda test, 5 mL of the hydroethanolic extract was mixed with 200 mg of magnesium turnings and 1 mL of HCl. The formation of orange color indicates the presence of flavonoids.

### 2.9. Assessment for the Presence of Coumarins

Thirty milliliters of hydroethanolic extract was acidified to pH 1 and concentrated to 10 mL in a water bath at 60°C. The residual extract was mixed with 5 mL of deionized water and transferred into a separation funnel with ethyl ether in 3 portions of 10 mL. The volume of the organic extract was reduced to 5 mL in a water bath at 60°C. Three drops of hydroethanolic extract were placed in two points of a premarked filter paper and left to dry and 1 drop of 1 N NaOH was added on each spot. The stains were covered with a coin and observed under UV light. A blue or yellow-green fluorescence indicated a positive reaction.

### 2.10. Assessment for the Presence of Anthraquinones

20 mL of hydroethanolic extract was brought to a boil under reflux for 15 minutes by adding 3 mL of 10% H_2_SO_4_. After cooling, the mixture was transferred to a separatory funnel along with 30 mL of distilled water to perform an extraction with 10 mL of toluene. The extract was concentrated to 10 mL and mixed with 10 mL of NaOH. The appearance of pink or red color indicated the presence of hydroxy-anthraquinones and naphthoquinones.

### 2.11. Assessment for the Presence of Sterols and Triterpenes

20 mL of hydroethanolic extract was evaporated to perform an extraction with dichloromethane. Extracts were concentrated (3 mL) and mixed with 2 mL of acetic anhydride and 3 drops of H_2_SO_4_. The development of blue-green color demonstrated the presence of steroids and/or triterpenes.

### 2.12. Assessment for the Presence of Anthocyanin and Saponin Glycosides

Three portions (5 mL each) of aqueous extract were neutralized with 5% KOH until a pH of 5.5 was reached. Changes in color indicated the presence of the neutralized portions of anthocyanin glycosides. To test for the presence of saponin glycosides, the remaining solution was stirred vigorously for 5 min. The appearance of persistent foam in the sample indicated the presence of saponins. This was further confirmed by adding 1% HCl to the sample.

### 2.13. Assessment for the Presence of Cyanogenic Glycosides

15 mL of the aqueous extract was transferred to a test tube. After adding 1 mL of 1 N H_2_SO_4_, a strip of picrosodium paper was secured inside the tube while it was in a water bath at 60°C for 30 minutes. The appearance of red color on the paper indicated the presence of cyanogenic glycosides.

### 2.14. Assessment for the Presence of Gums, Tannins, and Mucilage

Five drops of 10% basic acetate and neutral lead acetate were added to 2 portions of 5 mL of aqueous extract. The precipitate formation is indicative of the presence of gums, mucilages, and tannins. To assess the presence of tannins, 5 drops of 1% FeCl_2_ were added to 5 mL of the aqueous extract. Upon dark precipitate formation, 5 mL of the aqueous extract was transferred to a flat bottom flask to add 5 drops of 37% formaldehyde and 4 mL HCl. The mixture was left to reflux for 1 hour and when it cooled, it was filtered and washed with distilled water and alcohol. A few drops of 5% KOH were added to the material retained on the filter. The appearance of color indicated the presence of condensed tannins. The filtrate was then mixed with 10 drops of 1% FeCl_3_, the formation of a dark blue precipitate confirmed the presence of hydrolyzable tannins.

### 2.15. Assessment for the Presence of Amino Groups

After concentrating 10 mL of the aqueous extract at a temperature of 50°C, 5 drops of concentrated extract were dropped on a filter paper. After drying, the filter paper was sprayed with a solution of ninhydrin in butanol and heated at 90–100°C for 15 min. The appearance of blue-violet color indicated the presence of amino groups.

### 2.16. Assessment for the Presence of Volatile and Fixed Acids

10 mL of aqueous extract was acidified with 1 N H_2_SO_4_ and brought to a boil in a water bath. A pH strip was used to measure the acidity of the vapors produced during boiling. The acidic color indicates the presence of volatile acids. 20 mL of aqueous extract was transferred to a distillation flask along with 2 mL of 1 N NaOH. The mixture was left to reflux for 30 minutes and after cooling and acidifying with 1 N H_2_SO_4_ an extraction with ethyl ether was performed. The extracts obtained were filtered and evaporated to dryness. After heating the residue for 10 min at 100°C and adding 5 mL of 1 N NH_4_OH, it was filtered again. Three drops were transferred to a filter paper to obtain a spot of 1 cm in diameter. The paper was dried at 100°C for 10 minutes and treated with Nessler's reagent. The color development indicated the presence of fixed acids.

## 3. Results and Discussion

The results for the systematic phytochemistry analysis can be seen in Tables 3 and 5.

In this first set of assays, we obtained positive results for the presence of alkaloids and most of them precipitated in neutral or slightly acidic medium. It should be emphasized that these precipitates can also be caused by proteins, purines, alpha pyrones, certain coumarins, lignans, and phenolic hydroxyl compounds. Negative results with these reagents are indicative of the absence of alkaloids; however, the formation of precipitates can only be considered as probable presence thereof [24].

The acidity of the fruit was confirmed through the measure of pH of 5.5–6. This correlated with the results shown by Andrade et al. 1997 where it was calculated using the ratio of Brix (soluble solids content) and the acidity of the fruit yielding a low ratio, therefore showing its acidic potential.

It also presented a strong positive result for the presence of phenols, in contrast with the findings reported in a study by Andrade et al. (1997) in which a relatively low amount of phenolic compounds (14.4 mg) in 100 g of cubiu whole pulp was found [1]. According to Moreira and Mancini-Filho (2004), phenolic compounds are natural second class or secondary antioxidants [12]. This class of antioxidants acts by delaying the initiation of autooxidation by different mechanisms, for example, hydroperoxide decomposition to form nonradical species [13]. This finding confirms the potential antioxidant activity of cubiu.

In our study, we also found evidence of the presence of flavonic heterosides. Previous studies have shown that flavonoids exert several activities over different biological systems showing antimicrobial, antiviral, antiulcerogenic, cytotoxic, antineoplastic, antioxidant, antihepatotoxic, antihypertensive, hypolipidemic, anti-inflammatory, and antiplatelet activities. Flavonoids have also been found to increase capillary permeability and inhibit protein exudation and leukocyte migration [25]. These effects may be related to the inhibitory effect that flavonoids exert over different enzyme systems including hydrolases, isomerases, oxygenases, oxidoreductases, polymerases, phosphatases, proteins, and amino acid oxidases phosphokinases [26]. The presence of coumarins can be related to antioxidant activities, along with other possible mechanisms, such as anti-inflammatory, antispasmodic, antitumor properties, and interaction with several enzymes.

In the second set of assays (Table 5), the presence of anthocyanin heterosides, acids (volatile and fixed) and, in greater quantity, amino groups and gums, mucilages, and tannins was noted.

The anthocyanin or anthocyanin heterosides are substances belonging to the flavonoid family. These are pigments that give color to flowers, fruits, leaves, stems, and roots of plants [27]. In previous studies, anthocyanins were found to have an inhibitory action on lipid peroxidation [28] and a protective action over vascular epithelial cells against reactive oxygen species, principally to H_2_O_2_-induced loss of cell viability [29].

Gums are generally considered a pathological result due to physical injury suffered by plant tissues by the action of microorganisms or due to unfavorable conditions such as drought. Mucilage gums were detected by precipitation in tube assays. However, neither of both has antioxidant characteristics [30]. The amino groups were detected by the presence of purple coloring. They are compounds bound to an NH_2_ radical, structurally similar to the alkaloids but with no oxidative activity due to the gradual reduction of the radicals present in their structures [24].

**Figure 2:**
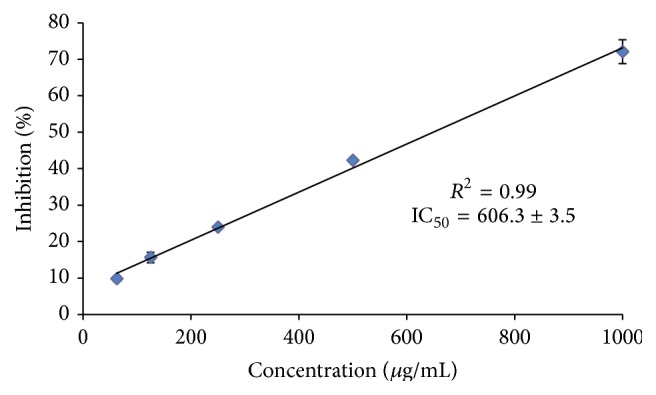
Colorimetric assessment of antioxidant capacity using in the DPPH assay. Values represent mean ± standard deviation of the mean of replicate readings (*n* = 3). The IC_50_ values denote the concentration of the sample, which is required to scavenge 50% of free radicals.

The antioxidant activity of the 70% hydroethanolic extract of cubiu was evaluated using* in vitro* assays: DPPH and ABTS. In this study, the sequestering capacity of the hydroethanolic extract of cubiu was expressed as the final concentration of extract required to inhibit oxidation of DPPH by 50% (Figure 2). The antioxidants in the extract react with DPPH, which is a stable free radical, and convert it into 2,2-diphenyl-1-picryl hydrazine. The degree of discoloration indicates the antioxidant potential of the extract. An extract that has a high antioxidant potential has a low IC_50_ [31]. Our results show that IC_50_ of cubiu was 606.3 ± 3.5 *μ*g/mL, a high value when compared with ascorbic acid (IC_50_ of 2.74 ± 0.3 *μ*g/mL), which used as a reference substance.

In a study comparing the antioxidant activity of native fruits, the lower IC_50_ values were obtained by gallic acid (1.38 mg/mL), ethanolic and aqueous pequi bark extracts (9.44 and 17.98 g/mL, resp.), ethanolic cagaita seed extract (14.15 mg/mL), and ethanolic extract of araticum seed and peel (30.97 and 49.18 mg/mL, resp.) [32]. In a study that evaluated the antioxidant activity of several brands of orange juice using the DPPH assay, it was found that the ones with activity were the orange file (66.24%) and the orange bahia (60.32%) [33]. However, it is difficult to compare the antioxidant activity of different samples because the authors used different dilutions of the samples to conduct the analysis, since each sample has a different antioxidant power. Furthermore, the ways to analyze and display the results are different. Stratil et al. (2007) reported that the comparison of results regarding the antioxidant capacity published in individual methods and among groups that use the same method is often problematic [34].

**Figure 3:**
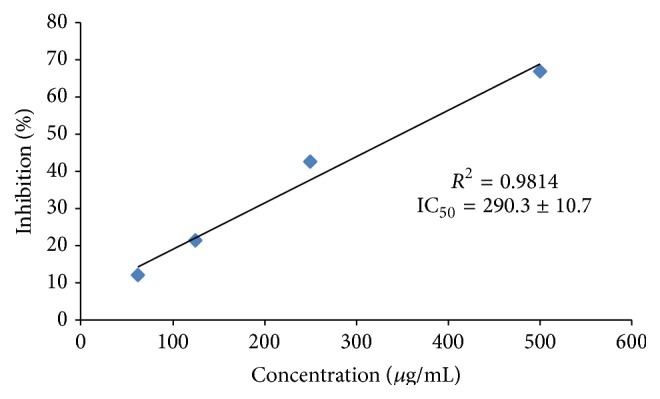
Results from the ABTS test. Values represent mean ± standard deviation of the mean of replicate readings (*n* = 3). The IC_50_ values denote the concentration of the sample, which is required to scavenge 50% of free radicals.

Regarding the results obtained by the ABTS method (Figure 3), the values obtained were 290.3 ± 10.7 *μ*g/mL. In a study performed in the Singapore market, the ABTS method was used to measure the Equivalent Antioxidant Capacity L-Ascorbic Acid (EACA) and the results were expressed in mg of ascorbic acid (AA) per 100 g of the homogenate containing the fruit extract. In this study, the fruit with the highest antioxidant capacity was sapodilla (3396 mg/100 g), followed by strawberry (472 mg/100 g), plum (312 mg/100 g), and carambola (278 mg/100 g) [35]. Dos Santos et al. (2008) evaluated the antioxidant capacity of commercial açai pulp using the ABTS method and expressing the results as TEAC (Trolox Equivalent Antioxidant Capacity). The TEAC of açai pulp ranged from 10.21 to 52.47 mM of Trolox/g sample. This confirms the difficulty of comparing the antioxidant activity reported in different studies since each one uses a different method of analysis [36].

The results obtained in this study were conflicting when compared to those presented in other studies. Nascimento and Pereira (2011) used the DPPH method and stated that cubiu extracts obtained from methanol fraction contained the highest antioxidant activity (between 100 and 200 mE BHT/g of freeze-dried material) [37], which explains the fact that in this study the antioxidant activity was found to be low since the hydroethanolic extract was used. Another interesting finding of the study is that, of all the extracts obtained from different parts of the fruit, the bark extract stood out with higher antioxidant activity, which is usually discarded in food preparations. Ledur et al. (2012) used the DPPH method to evaluate the antioxidant capacity of the 70% hydroethanolic extract of cubiu. The bark extract showed the lowest IC_50_ when compared to the seed and pulp extracts (278.7 mg/mL and 309.98 mg/mL, resp.) [38]. These values are lower than the results obtained in our study; however, they confirm the fact that cubiu has little antioxidant capacity.

## 4. Conclusion

To achieve the objective of the study, flour of cubiu (*Solanum sessiliflorum *Dunal) fruit was prepared and used for the preparation of the 70% hydroethanolic extract that was further analyzed using different phytochemical assays and antioxidant capacity tests. From the phytochemicals assays, the presence of phenolic compounds and flavonoids was noted. Both of these substances are recognized in the literature for their great antioxidant potential. However, despite confirmation of the presence of these compounds, cubiu extract showed a low antioxidant activity when compared to other fruits such as sapodilla, strawberry, orange, cherry [35], and many others known for their great antioxidant effect.

## Figures and Tables

**Table 1:** Chemical composition of cubiu (*Solanum sessiliflorum*) in 100 g of whole pulp [1–5].

Component	Villachica (1996) [4]	Pahlen (1977) [3]	Andrade et al. (1997) [1]	Yuyama et al. (1997, 1998) [2, 5]
Units (g)	89	91	93	90
Energy (kcal)	41	33	31	45
Proteins (g)	0.9	0.6	—	0.9
Lipids (g)	—	1.4	—	1.9
N-free extract (g)	—	5.7	—	4.7
Fiber (g)	0.2	0.4	—	0.9
Ashes (g)	0.7	0.9	—	0.9
Total sugars (%)	—	—	4.6	—
Reducing sugars (%)	—	—	3.9	1
Nonreducing sugars (%)	—	—	1.8	1
Soluble solids (°Brix) %	—	5	8	—
Citric acid (%)	—	—	0.8	—
Brix/acidity	—	—	5.9	—
Phenolics (mg)	—	—	14.4	—
Tannins (mg)	—	—	142	—

**Table 2:** Vitamin and mineral composition of *Solanum sessiliflorum* in 100 g of cubiu whole pulp according to several studies and percentage of the daily recommendation of the National Research Council (1989) [14].

Component	Villachica (1996) [4]	Pahlen (1977) [3]	Andrade et al. (1997) [1]	Yuyama et al. (1997, 1998) [2, 5]	%NRC
Ascorbic acid (mg)	4.5	—	13.9	—	15.3
Niacin (mg)	2.3	2.5	—	—	14.1
Carotene (mg)	0.2	0.2	—	—	—
Thiamine (mg)	0.1	0.3	—	—	15.4
Riboflavin (mg)	0.1	—	—	—	6.6
Calcium (mg)	16	12	—	—	1.2
Magnesium (mg)	—	—	—	23.7	7.5
Phosphorus (mg)	30	14	—	—	1.8
Potassium (mg)	—	—	—	385.4	19.3
Sodium (mg)	—	—	—	371	74.2
Copper (mg)	—	—	—	329	14.6
Iron (mg)	—	—	—	324	2.6
Zinc (mg)	—	—	—	157	1.1
Manganese (mg)	—	—	—	97	2.8

**Table 3:** Tests for the 70% hydroethanolic extract of the fruit *Solanum sessiliflorum* Dunal.

Compound classes assessed	Test results
Alkaloids	
Mayer reagent	−
Dragendorff reagent	++
Bouchardat reagent	−
Bertrand reagent	++
Reaction of confirmation	Dragendorff and Bertrand
Organic acids	++ pH = 5.5–6
Phenols	+++
Heterogeneous flavonoids	
Tauböck or Oxalo-boric reaction	+++
Pacheco reaction	−
Shinoda reaction	+
Coumarins	+
Anthraquinones	−
Sterols and Triterpenes	−

+++: strongly positive/++: positive/+: traits/−: negative.

**Table 4:** Reagent composition and color of precipitate for the different tests used to determine the presence of alkaloids on the 70% hydroethanolic extract of the *Solanum sessiliflorum* Dunal fruit.

RGA	Composition	Color of precipitate
(1) Mayer	Potassium-mercuric iodide	Orange
(2) Dragendorff	Bismuth nitrate	White
(3) Bertrand	Silicotungstic acid	White
(4) Bouchard/Wagner	Potassium triiodide	Brown

**Table 5:** Tests for the aqueous fruit extract of *Solanum sessiliflorum* Dunal.

Presence of chemical groups	Test results
Anthocyanin heterosides	++
Saponin glycosides	−
Cyanogenic glycosides	−
Gums, tannins, and mucilage	+++
Tannins	+
Amine groups	+++
Volatile acids	++
Fixed acids	+

+++: strongly positive/++: positive/+: traits/−: negative.
